# Supplementary material for: Antigenicity, stability, and reproducibility of Zika reporter virus particles for long-term applications
Source: PLoS Negl Trop Dis. 2020 Nov 18;14(11):e0008730. doi: 10.1371/journal.pntd.0008730 (PMC7673510; doi:10.1371/journal.pntd.0008730)
Supplement: S1 Table — MAb reactivities for each alanine scan mutant are expressed as a percentage of reactivity with wild-type ZIKV prM/E, with ranges (half of the maximum minus minimum values) in parentheses. Values for critical residues are shaded in gray. Values shown are the average of at least two replicate experiments. Data for anti-ZIKV MAbs A9E, ZIKV-116, and ZIKV-117 are also shown as comparative controls. (DOCX) [file pntd.0008730.s001.docx]

**Table S1. Epitope mapping data for MAbs LM-081, C8 and C10**

|  | **Test MAbs** | | | **Control MAbs** | | |
| --- | --- | --- | --- | --- | --- | --- |
| **Residue Mutation** | **C10** | **C8** | **LM-081** | **A9E** | **ZIKV-116** | **ZIKV-117** |
| R2A | 5 (2) | 29 (5) | 43 (6) | 84 (11) | 116 (23) | 70 (2) |
| R73A | 28 (5) | 56 (3) | 92 (1) | 106 (28) | 121 (13) | 53 (9) |
| G78A | 72 (8) | 9 (2) | 41 (12) | 81 (5) | 97 (10) | 18 (3) |
| R99A | -1 (2) | 0 (1) | 53 (5) | 61 (6) | 108 (14) | 53 (9) |
| N103A | -1 (1) | 0 (1) | 25 (9) | 59 (1) | 96 (20) | 35 (3) |
| G104A | 19 (2) | 8 (1) | 41 (4) | 55 (6) | 84 (4) | 35 (2) |
| G106A | 160 (20) | 72 (2) | 22 (5) | 112 (14) | 120 (16) | 140 (28) |
| G109A | 7 (-) | -1 (1) | 74 (6) | 73 (5) | 104 (7) | 45 (4) |
| L113A | 105 (5) | 2 (3) | 98 (17) | 92 (10) | 90 (7) | 61 (11) |
| V153A | 24 (9) | 73 (3) | 117 (30) | 116 (3) | 133 (15) | 117 (14) |
| R252A | 193 (43) | 12 (2) | 101 (62) | 112 (8) | 111 (11) | 156 (15) |
| K316A | -2 (3) | 1 (3) | 42 (12) | 75 (1) | 75 (0) | 103 (15) |
| I317A | 73 (10) | 111 (26) | 13 (5) | 85 (12) | 109 (7) | 122 (5) |
| K373A | 42 (7) | 24 (4) | 80 (19) | 58 (11) | 47 (0) | 74 (11) |

MAb reactivities for each alanine scan mutant are expressed as a percentage of reactivity with wild-type ZIKV prM/E, with ranges (half of the maximum minus minimum values) in parentheses. Values for critical residues are shaded in gray. Values shown are the average of at least two replicate experiments. Data for anti-ZIKV MAbs A9E, ZIKV-116, and ZIKV-117 are also shown as comparative controls.
