# Supplementary material for: Antigenicity, stability, and reproducibility of Zika reporter virus particles for long-term applications
Source: PLoS Negl Trop Dis. 2020 Nov 18;14(11):e0008730. doi: 10.1371/journal.pntd.0008730 (PMC7673510; doi:10.1371/journal.pntd.0008730)
Supplement: S2 Table — a TYMC: total yeast and mold count (limit of detection: 1 cfu/mL). b TAMC: total aerobic microbial count (limit of detection: 2 cfu/mL). c GenScript ToxinSensor Gel Clot Endotoxin Assay System (limit of detection: 1 EU/mL). d Sigma LookOut PCR kit, result indicated by absence/presence of specific PCR product. e Lonza MycoAlert kit, result interpreted according to manufacturer’s protocol. (DOCX) [file pntd.0008730.s002.docx]

**Table S2. Quality control tests and results for luciferase ZIKV RVPs**

| **Test** | **Result** |
| --- | --- |
| Bioburden (USP61) | < 1 cfu/mL TYMC*^a^* |
| Bioburden (USP61) | < 2 cfu/mL TAMC*^b^* |
| Endotoxin | < 1 EU/mL*^c^* |
| *Mycoplasma* (PCR) | Negative*^d^* |
| *Mycoplasma* (ELISA) | Negative*^e^* |

*^a^* TYMC: total yeast and mold count (limit of detection : 1 cfu/mL)

*^b^* TAMC: total aerobic microbial count (limit of detection: 2 cfu/mL)

*^c^* GenScript ToxinSensor Gel Clot Endotoxin Assay System (limit of detection: 1 EU/mL)

*^d^* Sigma LookOut PCR kit, result indicated by absence/presence of specific PCR product

*^e^* Lonza MycoAlert kit, result interpreted according to manufacturer’s protocol
