# Supplementary figures and images for: Antigenicity, stability, and reproducibility of Zika reporter virus particles for long-term applications
Source: PLoS Negl Trop Dis. 2020 Nov 18;14(11):e0008730. doi: 10.1371/journal.pntd.0008730 (PMC7673510; doi:10.1371/journal.pntd.0008730)

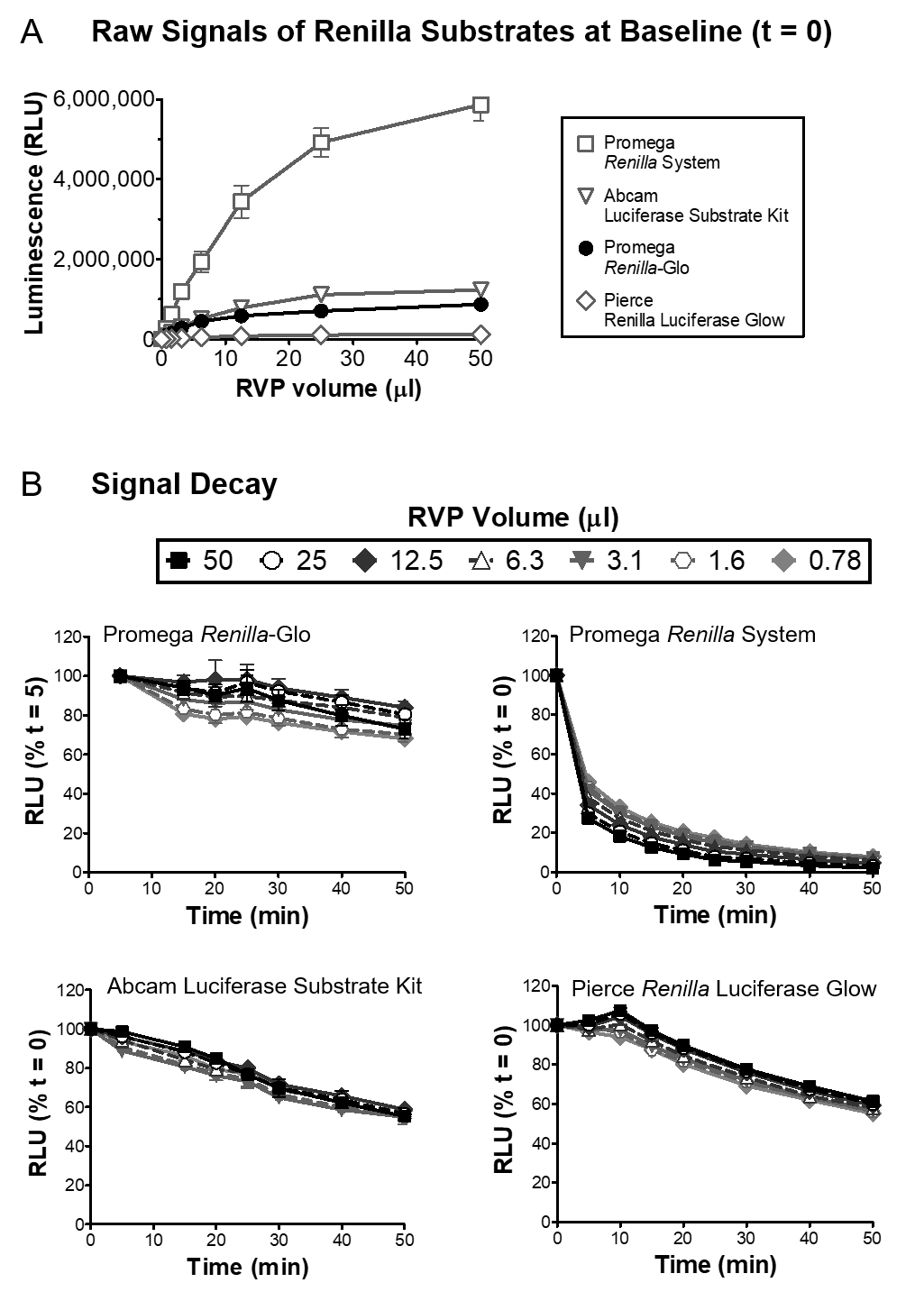

Supplement: S1 Fig — BHK-DC-SIGN cells infected with various volumes of luciferase RVPs were lysed and mixed with different luciferase substrates according to the manufacturers’ instructions. (A) Luminescence from individual samples (in relative luminescence units, RLU; n = 3, error bars with SD) was detected on an Envision plate reader after adding substrate. (B) Luminescence from individual samples was detected over time and plotted as a percentage of the signal at time 0 min (n = 3, error bars with SD). Luciferase assay reagents tested included the Promega Renilla-Glo Luciferase Assay System (E2710) (recommended), Promega Renilla Luciferase Assay System (E2810), Abcam Luciferase Reporter Assay Substrate Kit (ab228546), and Pierce Renilla Luciferase Glow Assay Kit (ThermoFisher 1616). For Promega Renilla-Glo (top left panel), luminescence was variable immediately after addition of substrate but stabilized after 5 min, so data was normalized to the 5 min time point rather than to 0 min. (TIF) [file pntd.0008730.s003.tif]

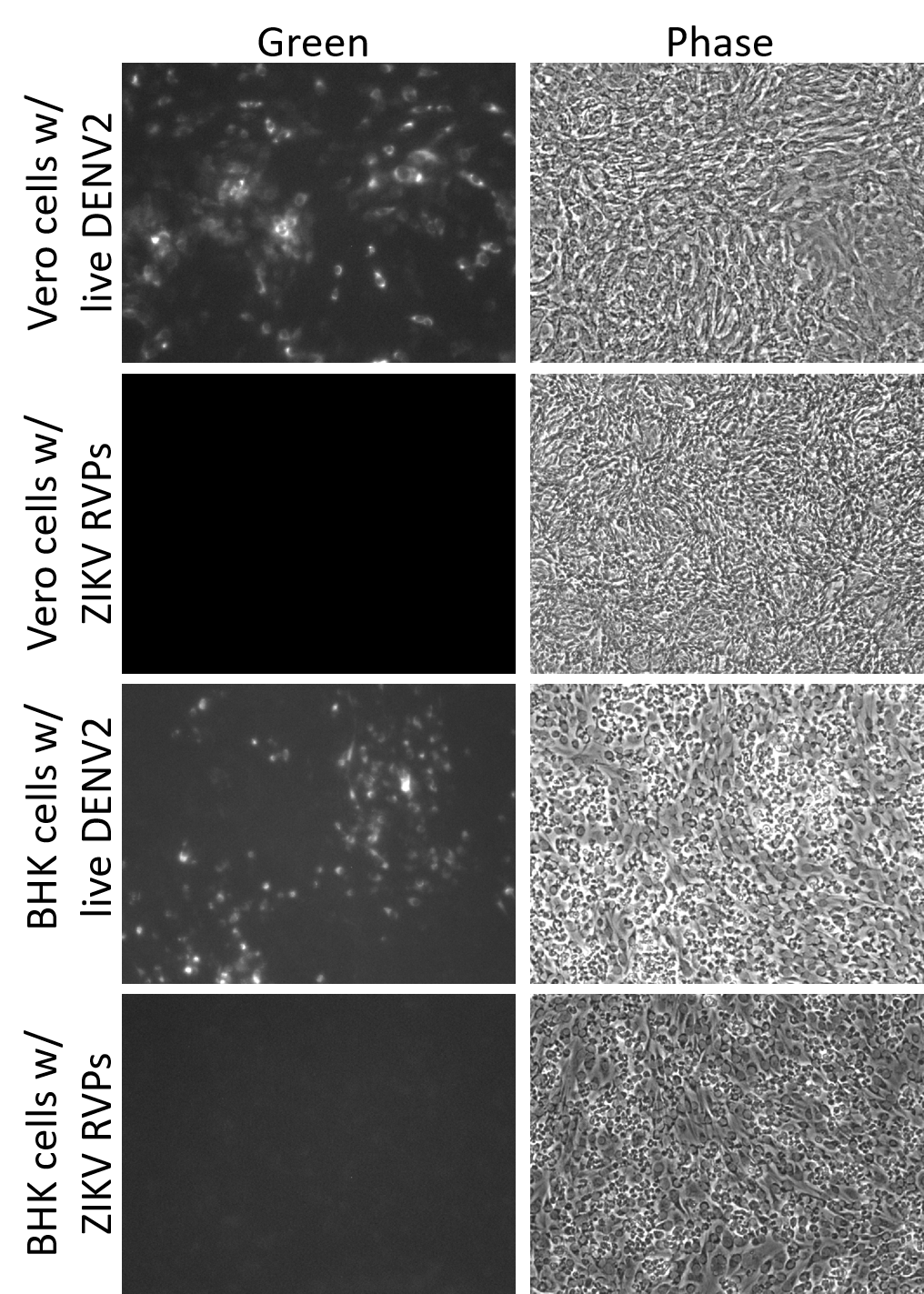

Supplement: S2 Fig — Vero or BHK DC-SIGN cells were seeded in 6-well tissue culture plates and grown for 1 d. Medium was replaced with ZIKV RVPs (1 mL/well) or live DENV2 virus in BHK Infection Medium. After 1 h at 37°C, 1 mL of medium was added and cells were grown for 4 d (37°C, 5% CO2). Cell monolayers were fixed, blocked, and stained for flavivirus E protein using MAb 4G2 (2 μg/mL) and goat anti-mouse Alexa Fluor 488. Fluorescence (Green) and phase-contrast microscopy (Phase) results are shown. (TIF) [file pntd.0008730.s004.tif]
